# The key role of miRNA in syndromic and sporadic forms of ascending aortic aneurysms as biomarkers and targets of novel therapeutic strategies

**DOI:** 10.3389/fgene.2024.1365711

**Published:** 2024-02-21

**Authors:** Sonia Terriaca, Roberto Monastero, Augusto Orlandi, Carmela Rita Balistreri

**Affiliations:** ^1^ Pathological Anatomy, Department of Biomedicine and Prevention, Tor Vergata University, Rome, Italy; ^2^ Section of Neurology, Department of Biomedicine, Neurosciences and Advanced Diagnostics, University of Palermo, Palermo, Italy; ^3^ Cellular, Molecular, and Clinical Pathological Laboratory, Department of Biomedicine, Neuroscience and Advanced Diagnostics (Bi N D), University of Palermo, Palermo, Italy

**Keywords:** epigenetics, miRNA, aortic aneurysms, thoracic aortic aneurysms, marfan syndrome, sporadic thoracic aortic aneurysms, biomarkers, therapeutic targets

## Abstract

Increasing evidence shows that epigenetics also plays a key role in regulating the pathogenetic mechanism of all types of aortic aneurysms. It is well-known that epigenetic factors modulate gene expression. This mechanism appears to be of interest especially knowing the relevance of genetic susceptibility and genetic factors in the complex pathophysiology of aortic aneurysms, and of sporadic forms; in fact, the latter are the result of a close interaction between genetic and modifiable lifestyle factors (i.e., nutrition, smoking, infections, use of drugs, alcohol, sedentary lifestyle, etc.). Epigenetic factors include DNA methylation, post-translational histone modifications, and non-coding RNA. Here, our attention is focused on the role of miRNA in syndromic and sporadic forms of thoracic aortic aneurysms. They could be both biomarkers and targets of novel therapeutic strategies.

## Introduction

**FIGURE 1:**
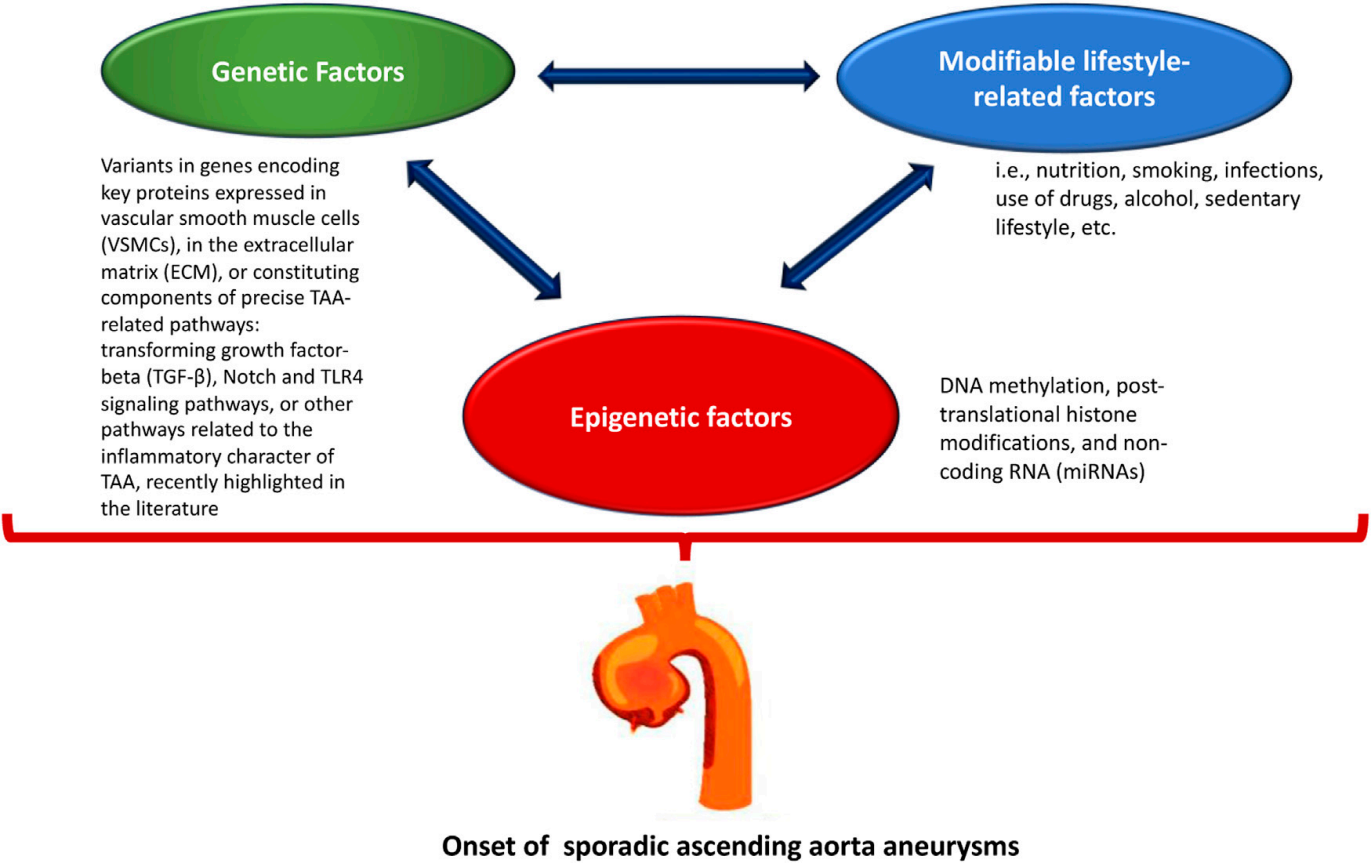
The onset of sporadic ascending aorta aneurysms is the result of the interaction of genetic, lifestyle, and epigenetic factors.

In recent years, many studies have investigated the role of epigenetic modulation in regulating the pathogenetic mechanism of all types of aneurysms (Chakraborty et al., 2023; Han et al., 2023; Rega et al., 2023; Tao et al., 2023). The regulation of gene expression appears to be quite interesting, especially considering the complex pathophysiological mechanisms underlying the sporadic forms of aortic aneurysms (Balistreri, 2015; Pisano et al., 2017; Saeyeldin et al., 2019), which are the result of a close interaction between genetic and modifiable lifestyle factors (e.g., diet, smoking, infections, drug use, alcohol, sedentary lifestyle, etc.) (Zhou et al., 2018; Downey and Aron, 2022; Zhou et al., 2022; Bararu et al., 2023; Chung, 2023; Crousillat et al., 2023; Dong et al., 2023; Salmasi et al., 2023) (Figure 1). However, male gender, age over 65 years, and a history of smoking are considered the main risk factors, although genetic factors are important in their development (Balistreri, 2015; Pisano et al., 2017; Hayder et al., 2018; Saeyeldin et al., 2019).

Here, our focus is on ascending thoracic aortic aneurysm (ATAA). ATAA is a typical pathological condition of the thoracic aorta, determined both by genetic and nongenetic factors. In general, an aneurysm occurs when the typical diameter of the artery increases by 50% (Balistreri, 2015; Pisano et al., 2017; Saeyeldin et al., 2019) and can lead to complications such us dissection and rupture.

ATAA is predominantly clinically silent and develops in the context of adverse degeneration and remodeling of the aortic wall, which can be inherited or acquired, and not associated with atherosclerosis when compared to abdominal aortic aneurysms (Balistreri et al., 2017; Waldron et al., 2023). This characteristic of the ATAA is linked to the different embryonic origin of this portion of the aorta when compared to the abdominal portion of the aorta. Accordingly, the aorta section from the ascending and aortic arch to the ligamentum arteriosus arises from neural crest precursor cells, while the section of the abdominal aorta arises from mesodermal precursor cells (Balistreri et al., 2017; Waldron et al., 2023). Furthermore, the different sections of the aorta show both heterogeneity in the density of extracellular matrix (ECM) microfibrils and the reactivity of vascular smooth muscle cells (VSMCs) to vasoactive growth factors, with downstream effects linked to the activation of different pathways, capable of determining a diverse susceptibility to the onset of aneurysms (Balistreri et al., 2017; Waldron et al., 2023). These characteristics make the ascending aortic tract more susceptible to vascular aging, particularly symptomatic in middle-aged individuals, and characterized by the development of a typical phenotype characterized having vascular remodeling and medial degeneration (Balistreri et al., 2017; Waldron et al., 2023). Precisely, such phenotype shows typical microscopic alterations, including endothelial dysfunction associated with increased oxidative stress, inflammatory innate responses followed by inflammatory cell innate and clonotypic infiltration into the aortic wall, VSMC apoptosis, aortic media degeneration, and elastin fragmentation and degradation. In the complex, these variations cause the decline of aortic elasticity. As a result, the aorta dilates and mechanical expansion forces are transferred to the ECM elements within the aortic wall and to the collagen (Balistreri et al., 2017; Waldron et al., 2023). In turn, this affects collagen within the tunica media to remodel, resulting in a stiffer and less compliant vessel. Increased aortic stiffness determines an augment in systolic and peak blood pressure, as well as an enhanced workload on the heart (Balistreri et al., 2017; Waldron et al., 2023).

ATAA can be divided into familial, in which one or more family members are diagnosed with ATAA, and nonfamilial or sporadic, where no other family members are affected (Brownstein, et al., 2018; Salmasi et al., 2023). Genetically determined ATAA can be considered the result of pathological variants in genes encoding key proteins expressed in VSMCs, extracellular matrix (ECM), or constituting components of precise ATAA-related pathways: transforming growth factor-beta (TGF-β), Notch and TLR4-signaling pathways, or other pathways related to the inflammatory character (Balistreri, 2015; Balistreri et al., 2017; Pisano et al., 2017) of ATAA, recently described in the literature (Postnov et al., 2021; Wortmann et al., 2021; Rodrigues Bento et al., 2022; Skotsimara et al., 2022). Genetically triggered ATAAs include Marfan (MFS), Loeys-Dietz (LDS), and vascular Ehlers-Danlos (vEDS) syndromes, or suffering from bicuspid aortic valve (BAV) syndrome (Asano et al., 2022; Carbone et al., 2023; Salmasi et al., 2023; Balistreri, 2018; Balistreri et al., 2019a and b). Alongside the genetic forms are the nongenetic forms of ATAA (i.e., sporadic ATAA), which are commonly diagnosed in older individuals, and particularly in those with vascular comorbidities, such as including hypertension (Balistreri, 2015; Balistreri et al., 2017; Pisano et al., 2017; Pisano et al., 2022a). However, sporadic ATAA forms are epidemiologically associated with the physiologic aging process, particularly vascular aging (as abovementioned), and in many individuals can occur even in the absence of other clinically evident features; such forms are also defined nonsyndromic ascending aortic aneurysm and dissection (nsATAAD) (Buffa et al., 2019; Salmasi et al., 2023). Currently, therapeutic treatments for all ATAAs, such as antihypertensive drugs, are not specific and poorly effective, so patients undergo surgery to remove the portions of the aorta that tend gradually but not rapidly to dilate. Moreover, most patients with ATAA eventually require surgical repair of the aneurysm, with mortality risk ranging from 1% to 5% for elective repair (Piano et al., 2021a; Asano et al., 2022; Nardi et al., 2022) and up to 12% for emergency surgery (Piano et al., 2021b; Asano et al., 2022; Nardi et al., 2022). Another typical feature of ATAA is the high phenotypic variability, which is well documented in all the forms of ATAA, but particularly in genetically triggered ATAA, in which the same pathogenic genetic variant can lead to different clinical manifestations (Salmasi et al., 2023). This emphasizes the uniqueness of each form of ATAA with a typical phenotype, which significantly affects the ability to predict prognosis and clinical outcomes, leading both clinicians and patients to uncertainty (Balistreri, 2018; Balistreri et al., 2019a; Balistreri et al., 2019b; Asano et al., 2022; Carbone et al., 2023; Salmasi et al., 2023).

Consequently, it is imperative to identify new factors that predict high-risk ATAA patients and to monitor response to therapy for personalized clinical management and to suggest more specific therapeutic strategies. In this regard, epigenetic factors are promising candidates that may help overcome the problems mentioned above (Kim and Stansfield, 2017; Knott and Doudna, 2018). Epigenetic mechanisms regulate gene expression without changing the DNA sequence (Jiang et al., 2020; Wei et al., 2020; Lareyre et al., 2023). The latter include DNA methylation, post-translational histone modifications, and noncoding RNA (Jiang et al., 2020; Wei et al., 2020; Han et al., 2023; Lareyre et al., 2023; Rega et al., 2023). In recent years, many studies have investigated the role of epigenetic mechanisms in ATAA pathophysiology (Chakraborty et al., 2023; Han et al., 2023; Rega et al., 2023; Tao et al., 2023).

In this review, recent evidence on the crucial role of microRNA in the pathogenetic mechanism of ATAA will be discussed. In addition, the role of omics technologies as therapeutic strategies for ATAA will be explored.

## Epigenetics: regulators of phenotypes

Before describing the epigenetic factors related to ATAA, it is necessary to briefly illustrate some concepts. Recent studies on “epigenome” and its characterization, which include endogenous mechanisms that can regulate gene expression independently of DNA sequence, have completely revolutionized our understanding of health and disease beyond traditional Mendelian genetics (Martínez-Micaelo et al., 2017; Margiotti et al., 2023). The term “epigenetics” refers to regulatory mechanisms of gene expression, ranging from DNA methylation, histone modification and non-coding RNA, such as microRNA (miRNA) and long non-coding RNA (lncRNA) (widely quoted in Jeffries, 2020; Zhang et al., 2020). Some studies have reported aberrant epigenetic modifications in the onset and progression of several human diseases (widely quoted in Jeffries, 2020; Zhang et al., 2020). Characterization of such modifications may allow facilitating or improving the understanding of disease mechanisms, as well as the development of disease-specific biological profiles. In particular, epigenetic mechanisms have been confirmed to be modifiable therapeutic targets (Conway et al., 2016). Therefore, they may provide enormous potential from a clinical perspective to improve diagnosis, monitoring, and treatment of a wide range of diseases, such as ATAA (Portelli et al., 2018). Therefore, the identification of aberrant epigenetic changes in ATAAs could allow a deeper understanding of their pathogenesis and provide an explanation for the high phenotypic variation observed among individuals. Furthermore, understanding these mechanisms could allow both the identification of new eligibility criteria for surgery and the development of new, more personalized therapeutic strategies.

## Epigenetic mechanisms: non-coding RNA_miRNA

In general, epigenetic mechanisms can be classified into three main categories: (I) DNA methylation; (II) post-translational histone modifications; (III) Non-coding RNA (Han et al., 2023). Among these, our focus is on non-coding RNA, particularly microRNAs (miRNAs) (Han et al., 2023). Non-coding RNAs act by affecting not directly chromatin architecture, but by demonstrating a major role in post-transcriptional regulation of gene expression (Li et al., 2023). The long non-coding RNAs (lncRNAs) constitute a large group of non-protein coding transcripts/protein non-coding transcripts longer than 200 nucleotides (Li et al., 2023). The mechanisms used by lncRNAs to regulate gene expression are unclear (Li et al., 2023). However, emerging evidence reports the ability of lncRNAs to control gene expression at multiple levels, including epigenetic control, transcription, RNA processing, and translation (Li et al., 2023). It has been observed that different lncRNAs are significantly enriched in the chromatin fraction and exhibit different functions: a) recruit chromatin-modifying complexes, including the Polycomb group (PcG) or the Trithorax group (TrxG); b) generate a repressive chromatin state or an active chromatin state; and c) influence gene expression in both cis or trans to distant target genes In addition, some lncRNAs can form RNA-protein complexes with transcription factors and control their localization and activity, as well as regulate gene expression (Mattick et al., 2023).

On the other hand, miRNAs are evolutionary conserved and constitute a class of non-coding RNAs of 18–22 nucleotides (O’Brien et al., 2018; Condrat et al., 2020). They regulate gene expression at the posttranscriptional level, generally interacting with the 3′ untranslated region (3′-UTR) of the target mRNA, resulting in its degradation and translational silencing (O’Brien et al., 2018; Condrat et al., 2020). The miRNAs are synthesized in the nucleus in a longer primary form (pri-miRNA) by RNA polymerase II. Subsequently, the pri-miRNA is processed by RNase III endonuclease, also known as *Drosha*, together with the cofactor *Pasha*, to produce the pre-miRNA of about 70 nucleotides. Next, the pre-miRNA is transported into the cytoplasm using GTP as a cofactor and is processed by another RNase III, called *Dicer*, generating a double-stranded miRNA of about 22 nucleotides. This product is incorporated into the multiproteic RISC (RNA-induced silencing complex) (O’Brien et al., 2018; Condrat et al., 2020). Within the RISC complex, a helicase ensures that only one of the duplex strands of the miRNA remains in the complex to control the post-transcriptional expression of target genes (O’Brien et al., 2018; Condrat et al., 2020). miRNAs have been reported to be critical regulators of cardiovascular function (Abubakar et al., 2023). Nevertheless, their role as biomarkers for the diagnosis of these diseases, including ATAA, and as therapeutic targets remains to be defined (Stougiannou, et al., 2023). Therefore, understanding and further investigating the role of miRNA in cardiovascular diseases may allow us to identify new potential prognostic factors and develop more specific therapeutic strategies for ATAA. Recent findings on the role of miRNAs in familial and sporadic ATAA will be described below.

## The regulatory role of miRNAs in familial ATAA

Patients with BAV or MFS have a higher risk of developing early aortic complications, such as ATAA and dissection, than individuals with a normal tricuspid aortic valve (TAV) (Maleki et al., 2019). In addition to the genetic factors that characterize familial ATAAs such as MFS and BAV, miRNAs have also been demonstrated to play an important role in the pathogenesis of those aortopathies (Maleki et al., 2019).

Maleki and coworkers have investigated the role of miRNAs in the pathogenesis of BAV aortopathies (Maleki et al., 2019). They used a systems biological approach, based on data of undilated aortas from BAV and TAV, revealing the key role of miR-200 family, which shows a strong association with the BAV signaling network. Aortic endothelial cells (ECs) appear to be the main source of miR-200c; ECs, explanted from BAV aortas, show lower miR-200c expression than with those obtained from TAV aortas. It has also been reported that the miR-200 family suppresses mRNA expression of Endothelial to mesenchymal transition (EndMT) transcription factors that are the zinc-finger E homeobox-binding transcription factors 1 and 2 (ZEB1 and ZEB2) (Brabletz and Brabletz, 2010; Hill et al., 2013). The lower expression of this miRNA in BAV has suggested a miR-200c dependent End-Mt process. ATAAs are known to be characterized by aortic remodeling with specific phenotypic changes in VSMCs and ECs that distinguish the various types of ATAAs (Maleki et al., 2019). In this regard, a study conducted by Pisano and coworkers showed that another miRNA is associated with the phenotypic changes that characterize BAV aortopathy (Pisano et al., 2022a). Precisely, they analyzed the involvement of the transcriptional factor ERG and its related pathways in the progression of BAV and TAV aortopathies by examining aortic tissues from individuals with BAV or TAV and affected or unaffected by ATAA. The authors of this work suggested the levels and functions of ERG protein, as miR-126-5P appears to be upregulated in aneurysmal aortic tissue samples from patients with BAV compared with those with TAV. Interestingly, this work has demonstrated the different aortic remodeling that characterize these aortopathies. In the ATAA endothelium of BAV cases, upregulation of miR-126-5P was found to be associated with downregulation of SMAD2/3 proteins compared with ATAA cases with TAV valve. Regarding the tunica media, BAV aneurysmal aorta samples showed obvious aortic calcification, whereas TAV aneurysmal aorta samples exhibited marked fibrosis. This work associates miR-126-5p with the different aortic remodeling of these two diverse aortopathies, ATAA with BAV and ATAA with TAV, and suggests this miRNA together with ERG as a biomarker to monitor ATAA progression (Pisano et al., 2022b).

As mentioned above, progressive aortic dilatation is characterized by increased aortic remodeling accompanied by activation of matrix metalloproteinases (MMPs) and downregulation of their inhibitors [tissue inhibitor of matrix metalloproteinases (TIMPs)] (Fedak et al., 2003; Nataatmadja et al., 2003). Wu and coworkers have recently reported that miR-17 regulates aortic dilation in BAV patients by downregulating the expression of TIMP-1 and -2 (Wu et al., 2016). These authors collected aortic tissue samples from patients with poorly and severely dilated BAV showing that upregulation of miR-17 is strong in less dilated aortic tissue and results parallel to the downregulation of TIMP-1, -2. Furthermore, VSMCs explanted from BAV aortas and transfected with a miR-17 mimic showed a downregulation of TIMP-1 and -2 expression and upregulation of MMP2 activity, with opposite effects compared with transfection with an inhibitor of miR-17, suggesting the crucial role of this miRNA in regulating TIMP-MMP balance. The upregulation of this miRNA in less dilated BAV tissues suggests that the regulation mediated by this miRNA begins in the early stages of aortic dilation. Therefore, its inhibition could prevent aortic dilation in these patients (Wu et al., 2016).

Other authors have reported different miRNA expression associated with the degree of medial degeneration (MD) in aortic and blood samples of BAV patients (Pisano et al., 2021a). The patient sample was divided according to the degree of MD into low-grade MD group (LGMD) and high-grade MD group (HGMD). The expression of selected miRNAs was first analyzed in the aortic tissues of patients and revealed that the expression of miR-718 and miR-122 was lower levels in the LGMD and HGD groups than the expression in the control group; in particular, miR-486 levels were found to be high in the HGD group. Subsequently the levels of these miRNAs were analyzed in blood samples from the same patients, showing that miR-122 was reduced in the two LGMD and HGD groups, and miR-718 was reduced only in the HGD group. In contrast, the expression of miR-486 was increased in both LGMD and HGD groups. Therefore, the authors suggested that miR-486 could be considered as a noninvasive biomarker of aortic wall degeneration (Pisano et al., 2021b).

It is well known that the onset and progression of BAV aortopathy are characterized by genetic, epigenetic, and hemodynamic factors, but few studies on this issue have been performed so far (Sophocleous et al., 2018; Balistreri et al., 2019a; Balistreri et al., 2019b). However, in a recent study it was shown that BAV is related to mutations in *ACTA2*, *FBN1*, *TGFBR2*, and *NOTCH1* genes (Giusti et al., 2017). Interestingly, Girdauskas et al. (2018) have studied the association between rare genetic variants and specific miRNA deregulations in BAV aortopathies. Specifically, the group of patients with BAV and aortic dilatation was divided into two additional subgroups, considering the presence or absence of rare genetic variants. Blood samples from both groups were analyzed for different miRNA expression, and the analysis showed that patients with *NOTCH1* variants showed significantly lower expression of circulating miR-145. This suggested a specific pathogenetic involvement of this miRNA in the pathogenesis of BAV and its correlation with rare genetic variants (Girdauskas et al., 2018).

As mentioned above, aortic samples from ATAA cases with BAV express lower levels of phosphorylated SMADs than those of ATAA and TAV (Pisano et al., 2022b). Regarding this aspect, Zhang and coworkers have identified a specific miRNA in exosomes from blood samples of patients with BAV, which was found to be associated with TGF-β signaling (Zhang et al., 2021). Zhang et al. (2021) discovered several exosomal miRNAs that are significantly deregulated in BAV. Among them, they have found significant upregulation of miR-423-5p, and prediction studies also reported that these miRNAs are involved in the regulation of TGF-β signaling. Therefore, they explored the potential target of miR-423-5p, identifying SMAD2 as gene target of miR-423-5p. Therefore, miR-423-5p negatively regulates TGF-β signaling, proving crucial for the onset and development of BAV disease and its complication, such as ATAA (Zhang et al., 2021).

On the other hand, MFS is characterized by hyperactivation of TGF-β signaling. Fibrillin-1 (*FBN1*) mutations, which characterize MFS, lead to altered sequestration of latent TGF-β (Dietz et al., 2005). The association between the pathogenesis of ATAA and dysregulation of TGF-β signaling has been the subjects of numerous investigations (Milewicz, 2012). Recently, studies have identified TGF-β responsive miRNAs that play critical role in cellular phenotypic modulation in ATAA and aortic development (Merk et al., 2012; Terriaca et al., 2023). Merk et al. (2012) have investigated the role of miR-29b, a miRNA known to regulate genes involved in apoptosis and ECM synthesis/deposition, in aneurysm formation in MFS cases. Specifically, they have analyzed the expression of miR-29b in the ascending aorta of MFS (Fbn1^C1039G/+^) and WT mice, revealing higher expression in the aorta of Fbn1^C1039G/+^ mice. The high expression of miR-29b was associated with increased aortic remodeling and decreased activation of nuclear factor kappa-light-chain-enhancer of activated B cells (NF-KB), a repressor of miR-29b and a factor suppressed by TGF-β. Administration of an NF-KB inhibitor increased the miR-29b levels, whereas TGF-β inhibition or losartan administration decreased miR-29b levels in Fbn1^C1039G/+^ mice, suggesting that TGF-β1 upregulates this miRNA. Finally, specific inhibition of miR-29b has been shown to prevent early aneurysm development, aortic wall apoptosis, and extracellular matrix deficiencies Merk et al. (2012).

Terriaca et al. (2023) recently described another TGF-β1-induced miRNA involved in the pathogenetic mechanisms of MFS (Terriaca et al., 2023). They reported the upregulation of miR-632 in aortic tissues of MFS ATAA compared with nongenetic ATAA tissues. The upregulation of miR-632 in MFS ATAA inhibited DNAJB6, inducing Wnt/β catenin signaling with End-Mt and exacerbation of fibrosis. In addition, TGF-β 1 treatments induced upregulation of miR-632 resulting in activation of the above processes. Therefore, the authors suggested that this miRNA might represent a novel therapeutic target and prognostic factors in the progression of MFS aortopathy.

As mentioned earlier, ATAAs are characterized by aortic remodeling and inflammation (Kessler et al., 2014; Welten et al., 2016). MFS ATAA showed severe morphological changes with increased fragmentation of the elastic lamina and infiltration of inflammatory cells (D’Amico et al., 2020). Zhang et al. (2022) have demonstrated that downregulation of miR-122 in human and mice MFS aorta causes upregulation of inflammatory cytokines and MMPs. In detail, these authors described that miR-122 is the most downregulated miRNA both in the aortas of Fbn1^C1041G/+^ and Fbn1^mgR/mgR^ mice, the latter used as severe mouse model. This downregulation was correlated with upregulation of chemokine (C-C motif) ligand 2 (CCL2), (Interleukin-1β) IL-1β and MMP12. In addition, they have also demonstrated that hypoxic conditions in cell and organ cultures reduced miR-122 expression. Hypoxia-inducible factors 1 (HIF-1α) inhibitors, used in both aortic smooth muscle cells and Fbn1^mgR/mgR^ mice, restored miR-122 expression and reduced elastin fragmentation, inflammatory infiltration, and aortic dilation. In addition, the authors have revealed molecular interactions between fibrillin-1 and miR-122. Therefore, this study showed the downregulation of miR-122, which is suppressed by fibrillin-1 deficiency and hypoxia, is responsible for the severe aortic morphological alterations and inflammatory infiltrations that characterize MFS ATAA (Zhang et al., 2022).

## The regulatory role of miRNAs in sporadic ATAA

In contrast to familial ATAA forms, the molecular and genetic mechanisms involved in the pathogenesis of sporadic ATAA, are not completely clear (El-Hamamsy and Yacoub, 2009). Generally, sporadic ATAAs have multifactorial etiology and are more common in older age than in familial ones (Shen and LeMaire, 2017). Sporadic ATAA share many features with familial ATAA, such as activation of TGF-β signaling, vascular remodeling, inflammatory cell infiltrate and fibrosis (Scola et al., 2014). Moreover, they are often asymptomatic and commonly detected incidentally during instrumental analysis, when the aortic dilation reaches a diameter that requires surgical treatment (Moushi et al., 2020). For those reasons, it is necessary to better understand the pathogenesis of sporadic ATAA to identify non-invasive biomarkers that predict aneurysm formation, and can be correlated to type, risk, or severity of disease (Moushi et al., 2020). miRNAs seem to be promising candidates as biomarkers for the early diagnosis or as therapeutic target for sporadic ATAAs (Moushi et al., 2020). Moushi et al. (2020) have investigated on the different miRNA expression in plasma samples of sporadic ATAA patients before and after surgery. miRNA expression profiling has revealed three miRNAs (hsa-miR140-5p, hsa-miR-191-5p and hsa-miR-214-3p), significantly upregulated in plasma samples derived from patients before surgery. Moreover, they have validated the expression of miRNA predicted targets, revealing *Myotubularin-related protein 4 (MTMR4)* as gene target of hsa-miR-140-5p and hsa-miR-214-3p, and *Phosphatase 1 catalytic subunit β (PPP1CB)* as gene target for hsa-miR-140-5p and hsa-miR-191-5p (Moushi et al., 2020). Those genes are involved in the TGF-β signaling pathway, having as well-recognized a critical role in ATAA, as largely mentioned above (Yu et al., 2010; Korrodi-Gregorio, et al., 2014). Being, however, a pilot study, the authors have suggested that the three miRNAs identified could be useful as biomarkers for sporadic ATAA but need further investigations. Gasiulė et al. (2019), have proposed other miRNAs as biomarkers for sporadic ATAA. Precisely, they have analyzed a panel of different miRNAs comparing tissue and plasma samples derived from sporadic ATAA patients and controls. For ATAA patients, plasma have been collected before and after surgery. The analysis has revealed 17 ATAA-specific miRNAs in tissue and plasma samples. Three of those ATAA-specific plasma miRNAs, miR-155b-5p, miR-122-3p and miR-23b-5p, result able to restore their expression to normal level after surgery, indicating their specific correlation with the pathology. Moreover, some of those specific miRNAs seems to be involved in TGF-β pathways, and precisely they are associated with TGF-β receptors, SMADs and Krueppel-like factor 4 (KLF4), whose molecules have been demonstrated to be upregulated in ATAA tissues (Gasiulė et al., 2019). Therefore, these miRNAs are key component in TGF-β signaling, and deepening their role could be useful to identify them as potential candidates for prognostic biomarkers of sporadic ATAA.

Overall, those studies have identified miRNAs as key regulators of the pathogenetic mechanisms of both sporadic and familial ATAA forms. Further studies are needed to evaluate these miRNAs in the blood samples and correlate their expression to the disease progression and severity, though the evaluation of clinical and instrumental data, to use them as non-invasive prognostic ATAA biomarkers. Moreover, *in vitro,* and *in vivo* studies could be useful to assess the induction or inhibition of those deregulated miRNA to develop new therapeutic strategies that counteract the onset/progression of those aortopathies. A summary of all mentioned miRNA and their role in sporadic and familial ATAA is reported in Table 1.

**TABLE 1:** Summary of deregulated miRNA in familial and sporadic ATAA.

miRNA	Type of deregulation	Samples analyzed	Genes associated	Pathogenetic mechanism	Type of ATAA	References
miR-200c	Downregulation	Human Aortic tissues and explanted endothelial cells	ZEB1 and ZEB2	End-Mt	BAV	Maleki et al. (2019)
miR-126-5p	Upregulation	Human Aortic tissues	SMAD2/3	Calcification	BAV	Pisano et al. (2022a)
miR-17	Upregulation	Human Aortic tissues	TIMP-1, -2	ECM breakdown and aortic dilation	BAV	Wu et al. (2016)
miR-486	Upregulation	Human Aortic tissues and blood samples	Not specified	Aortic remodeling, apoptosis	BAV	Pisano et al. (2021a)
miR-145	Downregulation	Human blood samples	rare NOTCH1 variants	VSMC proliferation and phenotypic switch	BAV	Girdauskas et al. (2018)
miR-423-5p	Upregulation	Human blood samples	SMAD2	Dysregulation of TGF-β signaling, angiogenesis	BAV	Zhang et al. (2021)
miR-29b	Upregulation	Mice Aortic tissues	NF-*k*B, caspase-3, caspase-9,Mcl-1 and Bcl-2	Aortic wall apoptosis and ECM abnormalities	MFS	Merk et al. (2012)
miR-632	Upregulation	Human Aortic tissues	DNAJB6	End-Mt and fibrosis	MFS	Terriaca et al. (2023)
miR-122	Downregulation	Human and mice aortic tissues	CCL2, IL-1β and MMP-12	Inflammatory responses and ECM remodeling	MFS	Zhang et al. (2022)
miR140-5p, miR-191-5p and miR-214-3p	Upregulation before surgery	Human aortic tissues and blood samples before and after surgery	MTMR4 and PPP1CB	Dysregulation of TGF-β signaling	Sporadic	Moushi et al. (2020)
miR-155b-5p, miR-122-3p and miR-23b-5p	Up and downregulation	Human aortic tissues and blood samples before and after surgery	TGF-β receptors, SMADs and KLF4	VSMC phenotype switch	Sporadic	Gasiulė et al. (2019)

**Abbreviations**: ZEB1/ZEB2, zinc-finger E homeobox-binding transcription factors; BAV, bicuspid aortic valve; End-Mt, endothelial to mesenchymal transition; TIMP-1, -2, tissue inhibitor of matrix metalloproteinases 1-2; ECM, extracellular matrix; VSMC, vascular smooth muscle cell; TGF-β, transforming growth factor β; MFS, marfan syndrome; NF-KB, nuclear factor kappa-light-chain-enhancer of activated B cells; Mcl-1, induced myeloid leukemia cell differentiation protein; Bcl-2, B-cell lymphoma 2; DNAJB6, DnaJ heat shock protein family (Hsp40) member B6; CCL2, chemokine (C-C motif) ligand 2; IL-1β, interleukin-1β; MMP-12, matrix metalloproteinase-12; MTMR4, myotubularin-related protein 4; PPP1CB, phosphatase 1 catalytic subunit β; KLF4, krueppel-like factor 4.

## Omics combined technologies in ATAA as newest generation treatments

Currently, the technological progresses and the reduction of their costs have allowed deep investigations of the pathogenetic mechanisms of many diseases, including aneurysms, for developing newest generation treatments (Lareyre et al., 2023; Rega et al., 2023). In particular, the researchers are applying integrative multi-omics which appear to be necessary and show the potential to provide a greater quantity of information, ranging from the original causes of disease to the functional consequences or relevant interactions, and to clarify the physiological and pathophysiological role of miRNAs (Lareyre et al., 2023; Rega et al., 2023).

**FIGURE 2:**
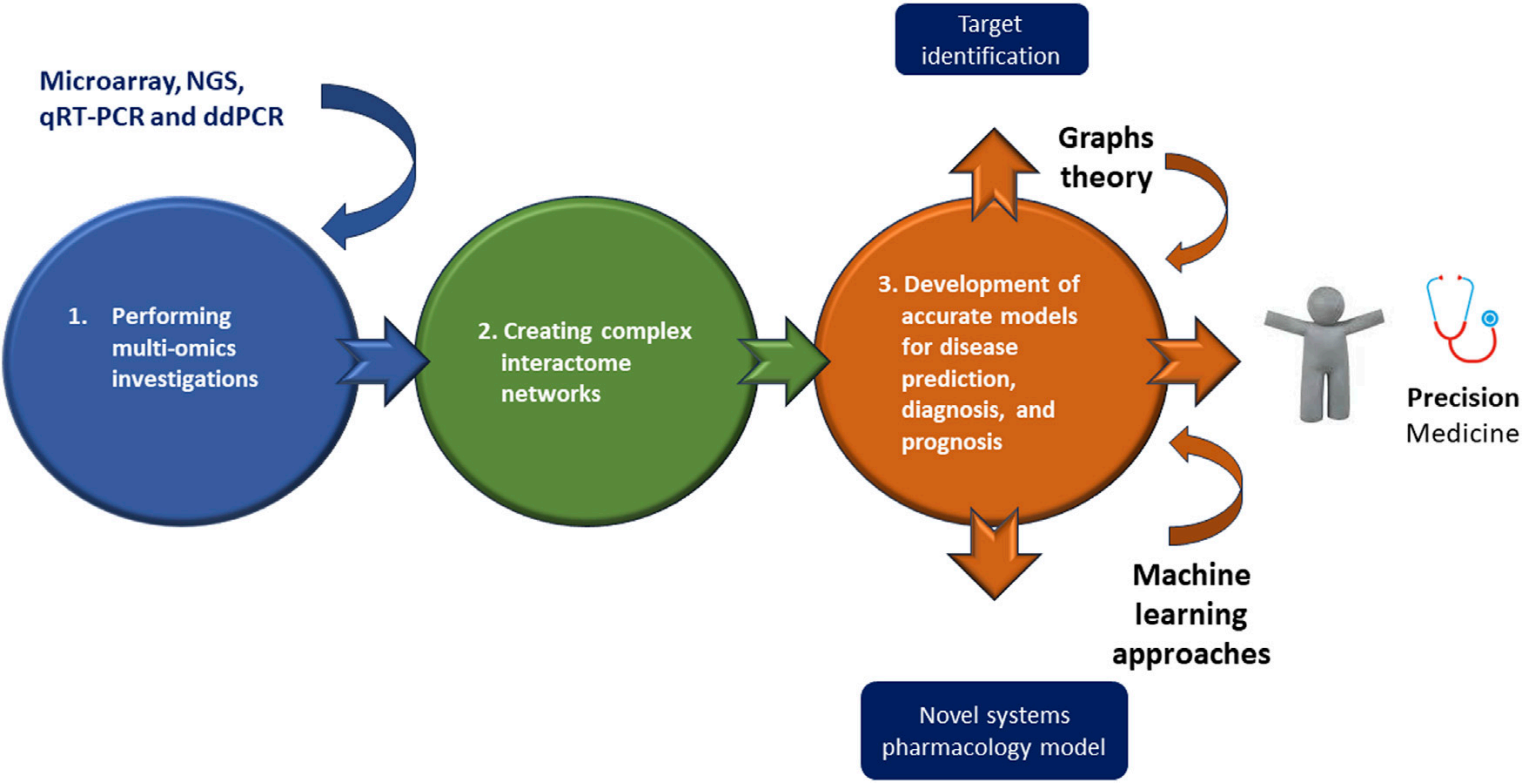
Omics combined technologies in ATAA as newest generation treatments. Executing multi-omics investigations may permit of identifying and creating complex interactome networks useful for developing accurate models for disease prediction, diagnosis, and prognosis thanks a graphs theory or machine learning approaches, or better for a personalized medicine, “precision medicine.”

However, while on one side, multi-omics approaches may appear as innovative strategies useful to interpret the mechanistic details of a disease, on the other, they make very difficult the analysis of related data. Accordingly multi-omics investigations typically depend on both creating complex interactome networks and developing precise models for disease prediction, diagnosis, and prognosis, by using graphs theory and machine learning approaches, respectively (Recanatini and Menestrina, 2023) (Figure 2). Both analyses constitute a very serious challenge (Recanatini and Menestrina, 2023), because firstly the interactions cannot be causal, since most links must be estimated through correlations or co-expressions; second, it is necessary to explore interactions between thousands or millions of entities (e.g., genes, epigenetic factors, proteins, metabolites, etc.) and this requires a huge amount of computational resources (Lareyre et al., 2023; Rega et al., 2023). Regarding machine learning analysis, the first issue is data harmonization and dimensionality (Lareyre et al., 2023; Rega et al., 2023). Second, it is necessary to combine databases with different observations and characteristics; third, classification models need to be combined with a huge number of features in many samples (Lareyre et al., 2023; Rega et al., 2023).

Therefore, integrative multi-omics approaches constitute tools for obtaining new relevant information from intricate molecular scenarios, although they present several difficulties, as mentioned above. Consequently, new studies combining multiple omics approaches on ATAA are strongly encouraged to identify key players in its pathogenesis and propose effective therapeutic strategies. Here, we propose the integrated use of liquid biopsy, i.e. peripheral blood, with omics methodologies, including microarray, NGS, qRT-PCR and ddPCR, to enable the identification of circulating miRNAs related to all stages of ATAA management and its prevention. Both microarrays and next-generation sequencing (NGS) can evaluate the genome-wide profile of many circulating nucleic acids, DNAs and RNAs, such as miRNAs. However, NGS presents higher sensitivity and ability to discover new RNA-based biomarkers. Quantitative reverse transcription PCR (qRT-PCR) can be used for the quantification of specific RNA species in fluids. If very high sensitivity is required, droplet digital polymerase chain reaction (ddPCR) can be used for absolute quantification of circulating miRNAs at the single-molecule level. Circulating miRNAs can be used as biomarkers for diagnosis, prognosis, and outcomes, as well as for its prevention, providing relevant information on the characteristics of ATAA, which correspond to unique disease phenotypes in each patient. On the other hand, Wang, and colleagues (Wang et al., 2007) have recently reported that informative gene expression signatures in peripheral blood cells can describe ATAA status and subtypes. Information on transcriptional programs in peripheral blood may lead not only to the identification of miRNAs, as biomarkers, but may also provide insight into pathways involved in the development of ATAA and highlight potential targets for therapeutic intervention. At the same time, the use of other methods, such as those of genetic engineering, such as the clustered regularly interspaced short palindromic repeats-associated protein 9 (CRISPR/Cas9) Editing and Base Primers (Knott and Doudna, 2018) can be used to correct hereditary diseases, such as familial forms of ATAA, caused by single point mutations in the nucleotide sequence of specific genes, such those abovementioned. The CRISPR/Cas technique can also be used to modify immune cells *ex vivo* and reintroduce them into patients, improving their efficiency in limiting inflammation, as in the case of sporadic ATAA form. Below is a brief description and applications of CRISPR/Cas9 editing and Base Primers.

## CRISPR/Cas9 editing and base primers

Based on the evidence reported above, we propose that a strong genetic editing technique might be a solution for reversing the onset and progression of familial forms of ATAA, as well as the integration of gene-editing techniques with nanotechnology (Dong et al., 2022; Testa and Musunuru, 2023). Therefore, the CRISPR/Cas9 system and techniques involved in the chemical modification of RNAs are emerging, and are also being incorporated into nanoparticles (NPs) for studying diseases (Dubey and Mostafavi, 2023), such as ATAA, and/or investigating potential disease corrections through restoration (Testa and Musunuru, 2023). The CRISPR/Cas9 system is substantially applied for both editing the genome of zygotes, thereby generating genetically modified animal species, and treating human diseases such as aneurysms (Dubey and Mostafavi, 2023). Furthermore, the development of cautious and efficient treatments is developing thanks to releasing of the CRISPR/Cas9 system into body tissues and targeting specific cells, including ECs or VSMCS (Dubey and Mostafavi, 2023).

Data in the literature suggest several types of delivery systems that enable genome editing. A delivery system with optimal efficacy in in vivo genome editing is based on the use of recombinant adeno-associated virus (AAV), but may cause adverse effects (Dubey and Mostafavi, 2023). Therefore, lipid NPs represent an alternative (Escalona-Rayo et al., 2023). For example, lipid NPs have been used to target macrophages in mice using the CRISPR/Cas9 system, although only a limited percentage (20%) of genome editing was observed (Escalona-Rayo et al., 2023). Therefore, it is a challenge to induce robust genome editing in target tissues, such as the aorta. However, a study was conducted on ECs, extracted from the lung, heart, aorta and peripheral vessels of adult mice, using a particular delivery system (Balistreri, 2022). Precisely, poly (ethylene glycol) methyl ether-block-poly(lactide-co-glycolide) (PEG-b-PLGA; PP)-based NPs have been used as vascular delivery. The obtained results demonstrated that polyethyleneimine (PEI)-formulated PP NP-mediated delivery of all-in-one CRISPR plasmid DNA expressing Cas9 under the control of the human CDH5 promoter and a guide RNA (gRNA) driven by the U6 promoter, resulted in highly efficient genome editing in ECs of various vascular locations with a single administration. A reduction in protein expression of approximately 80% in ECs was achieved. This approach could allow us to effectively modulate or eliminate the expression of genes that encode proteins strongly associated with phenotypic changes linked to the development of the aneurysm and its complications. Today, new forms of CRISPR-based technique have been developed. They are reported to be able to perform gene editing without the need for a DNA double-strand break (DSB), including base editors, master editors, and RNA-targeting CRISPR-associated protein (Cas) 13 effectors (widely cited in Balistreri, 2022). These CRISPR variants can potentially be integrated with NPs, although some issues require them to be fully optimized. Therefore, further studies are needed.

## Conclusion

The last two decades have highlighted a continuous increase in investigations with the main objective of improving knowledge of the molecular mechanisms linked to the pathophysiology of ATAA. The evidence described here documents the central role of epigenetic mechanisms, such as miRNAs, in the pathogenesis and progression of ATAA. Future investigations should be encouraged to better understand the specific miRNA deregulation associated with this pathology. Furthermore, specific study designs must be considered, as a significant number of studies have examined whole aortic tissue samples rather than specific cell types from aneurysmal or non-aneurysmal specimens. Furthermore, pharmacological interventions could be developed to reprogram a “diseased” epigenetic landscape, to be used as a therapeutic target for the treatment of such aortopathies. Understanding the entire structure of chromatin has led to the development of specific molecule, omics, and genetic engineering techniques, such as those mentioned above, capable of altering chromatin accessibility by enhancing or repressing epigenetic marks on DNA/histone complexes. The challenge for future investigations remains how to achieve tissue-specific modulation of chromatin with an efficient and specific delivery system, since systemic inhibition or activation may result in harmful side effects. NPs represent a solution, although several issues need to be resolved. With future research, epigenetics could serve as a useful framework through which to gain new insights into the pathogenesis of ATAAs and develop personalized treatments and management of ATAAs (Figure 2).
